# Notes and News

**Published:** 1901-05-18

**Authors:** 


### NOTES AND NEWS.

Dr. George Turner has been appointed Medical Officer of Health for the Transvaal. Previous to this appointment, Dr. Turner held a similar post in Cape Colony, and latterly has been acting in a temporary capacity for the Transvaal.

An outbreak of typhoid and scarlet fever has taken place in Bethnal Green and Shoreditch. The infection is regarded by the authorities as originating from a supply of milk coming from Staffordshire, and this has been stopped.

The foundation of a cottage hospital at Sutton, Surrey, has been laid, which is the gift of Mr. Passmore Edwards, Mr. R. Collingwood Forster, of Sutton, giving the site. Mr. Sharp, of Queen Anne's Gate, is the architect. The building will accommodate twelve beds, distributed over four wards, placed in wings on either side of the administration building.

The decision has been given in the House ot Lords on the Bill promoted by the Governors of St. Bartholomew's Hospital respecting the site of Christ's Hospital. It will be remembered that St. Bartholomew's Hospital has always expected to have the option of acquiring the ground to be abandoned by the Almoners of Christ's Hospital on reasonable terms. But the Almoners have in the meantime received offers to the amount of upwards of £700,000 for their five and a half acres of ground, and they repudiated the understanding with St. Bartholomew's. The decision now arrived at in the House of Lords provides for the whole matter to be referred to arbitration.

In the ambulance section of the Naval and Military Exhibition, to be opened at the Crystal Palace on the 23rd inst., the various hospital arrangements adapted to the requirements of a campaign will be one of the principal features. There will be a model of the Princess of Wales's Hospital Ship, showing the inner working of such relief transports. There will also be one of the carriages of the Princess Christian Hospital Train, fitted up in every way like those of the hospital train used in South Africa. Model Field and Base Hospitals with a model Receiving Station and Field Kitchen are also included as well as a Field Post Office and Field Telegraphs, in which it is hoped arrangements can be made for carrying on souvenir Post Office work, as was done at South Kensington and the Guildhall on the occasion of the Jubilee of the Penny Post.

We understand that Dr. F. Waldo, who retired from his candidature for the post of Medical Officer of Health for the City of London in favour of Dr. Collingridge, will become a candidate for the coronership of the City when it becomes vacant. There can, we think, be no doubt that the office of coroner should be held by a medical man, but when, as in this case, the candidate is both a medical man and a barrister, he has a double claim. The same may be said in regard to Professor W. R. Smith, who is likely to become a candidate.

The present is the season when annual reports are as plentiful as buttercups. Like the circulars of the halfpenny post their name is legion, and for this reason the ingenuity of the more active and intelligent managers of institutions is ceaselessly occupied in giving their report an attractive appearance. This year a revolution is taking place in the covers of these annual reports. Formerly, like the maiden aunt, they were draped in sombre hue. Nowadays we are threatened with every colour in the spectrum, and the latest specimen to hand this morning appears in a brilliant metallic green embossed cover, which has the merit from its aggressive appearance of exciting controversy in the family circle as to whether or not the colour is at all suitable or attractive. Our congratulations to the Hon. Sydney Holland on this latest novelty in charity advertisement.

The establishment of the New Richmond Surgical Hospital in Dublin confers a benefit, not only upon the patients who will receive treatment in it, but also upon the population in general, as in order to erect it on its present site, a very insanitary area of Dublin was swept away. The hospital, which was recently opened by the Lord Lieutenant, forms one of a group of three hospitals, the survivors of an ancient charity called the House of Industry Hospitals. These hospitals have a certain income, but it was necessary to appeal to the charitable for building purposes, and for the Richmond Hospital £30,000 was collected. Mrs. Hamilton, widow of the late well-known surgeon Mr. John Hamilton, contributed £6,000; Mr. L. Higgins' executors gave £6,000; the trustees of the Weir Legacy gave £18,000; and Lord Iveagh and others contributed generously. The hospital is governed under a special Act of Parliament.

It has often troubled the anxious mind of a zealous secretary how to keep the peace and promote satisfaction when a great personage honours the occasion of the annual meeting with his or her presence. On such occasions the first row of chairs is apt to be invaded by all and sundry. The latest device is to intimate to the governors that the front row of chairs will be reserved for the Council. Another difficulty occurs when the patrons and titled supporters, or some of them, who have attended in former years, are not desired particularly to attend such a large meeting. Then a post-card containing the formula, "the front row of chairs will be reserved for members of the Council," is felt to effectually annihilate any intention which this latter class of governor may have had of putting in an appearance. The intimation is as insinuating as it is amusing, and we shall be curious to know how far it will succeed in connection with the approaching annual meeting of an association which has made most wonderful progress under an able honorary secretary, to whose zeal and persistence its great success is mainly due.

